# Investigation of the Use of Cu as Top Electrode in Polymer Solar Cells

**DOI:** 10.3390/polym18020232

**Published:** 2026-01-16

**Authors:** Semih Yurtdaş

**Affiliations:** Department of Energy Systems Engineering, Faculty of Engineering, Karamanoğlu Mehmetbey University, 70100 Karaman, Türkiye; syurtdas@kmu.edu.tr

**Keywords:** cost-effective, Cu, polymer solar cell, stability

## Abstract

Reducing electrode-related costs is an important step toward the large-scale commercialization of polymer solar cells. In this study, Cu is investigated as a low-cost top electrode in inverted polymer solar cells with the architecture ITO/ZnO/P3HT:PCBM/MoO_3_/Cu. The fabricated devices achieved a maximum power conversion efficiency (η) of 2.86%, with an open-circuit voltage (V_oc_) of 610 mV, a short-circuit current density (J_sc_) of 6.90 mA cm^−2^, and a fill factor (FF) of 68%. Long-term stability tests were carried out over a period of 12 weeks under glovebox, desiccator, and ambient room conditions, during which efficiency decreases of 23%, 53%, and 78% were observed, respectively. Structural and spectroscopic analyses suggest that device degradation is closely associated with O_2_- and moisture-induced effects on the Cu electrode. The results demonstrate that Cu can be effectively employed as a top electrode in polymer solar cells under controlled environmental conditions, highlighting its potential as a cost-effective electrode material for polymer solar cell applications.

## 1. Introduction

One of the indicators of development in a given society is energy production and consumption [1]. According to 2022 data from The International Energy Agency, 70% of the world’s electricity production comes from fossil resources [2]. However, fossil fuels are decreasing globally day by day [3]. In addition, the gases resulting from the burning of fossil fuels cause the greenhouse effect [4]. It has been determined that the average temperature of the Earth has increased by 1.4 °C due to an increase in CO_2_ concentration in the atmosphere in the last century [5]. It has been reported that if fossil fuel consumption continues at the same rate, the temperature of our planet will rise significantly, and this increase will lead to major catastrophes [6,7]. Renewable energy technologies are essential for reducing greenhouse gas emissions and tackling global warming by replacing conventional energy sources [8,9].

Photovoltaic energy is a promising alternative to electricity generation from fossil fuels [10]. Considering that the sun is a clean source of energy that is renewable and inexhaustible in terms of its lifespan, it is evident that photovoltaic energy will be the most important energy source in the future [11,12,13,14]. Among the various ways to harness photovoltaic energy, polymer solar cells attract great interest due to their low cost, light weight, potential for large-scale production, applicability to flexible surfaces, suitability for solution processes, and environmental friendliness [15,16,17,18,19,20,21].

To briefly mention the working principle of a polymer solar cell, an electron–hole pair (exciton) is formed by the absorption of a photon in the active layer. Excitons formed near the interface of the donor and acceptor molecules are separated into free electrons and hole charge carriers at the interface, and the hole charge carriers are transferred to the donor-type layer and the electrons to the acceptor layer. Then, the separated charge carriers are collected over the contacts [22,23]. The important point here is that the energy levels of the materials used are compatible with each other.

When the literature is examined, it is seen that metals such as Al, Ag, and Au are used as top contacts for polymer solar cells [24,25,26,27,28,29,30,31,32,33]. However, no study has been found on the use of Cu for polymer solar cells as top electrodes. The work function of Cu (4.65 eV) [34,35] is very close to that of Ag (4.7 eV) [36]. There are no problems in the work performed with Ag [26,36,37]. Therefore, it is predicted that the hole charge carriers from the MoO_3_ layer can be efficiently collected with Cu instead of Ag. In addition, the motivation of this study was created by considering the low cost of Cu [38,39]. Furthermore, the resistivity and electrical conductivity values of the metals frequently employed in the literature, including Al, Ag, Au, and Cu, are provided in Table 1. Based on this comparison, Cu exhibits the second-highest electrical conductivity after Ag, suggesting that Cu represents a promising electrode material not only due to its similar work function and cost-effectiveness, but also owing to its high electrical conductivity.

Some of the studies on the subject are briefly mentioned below. In a study conducted by Kumar and Balachander in 2016, they fabricated solar cells with the structure ITO/ZnO/P3HT:PCBM/MoO_3_/Metal using Al, Ag, and Au as the metals. They reported achieving efficiencies of 1.77%, 1.154%, and 0.736% for the devices with Al, Ag, and Au, respectively [41].

Kang et al. investigated the use of Au, Ag, and Cu as bottom electrodes instead of ITO. In devices produced with (ITO, Au, Ag, Cu)/PEDOT:PSS/P3HT:PCBM/LiF/Al structure, 2%, 1.96%, 2.06%, and 2% efficiency values were obtained for ITO, Au, Ag, and Cu bottom electrodes, respectively [42]. This study explored the use of Cu as a conductive layer instead of ITO, rather than as top contact.

Behrouznejad et al. investigated the use of different metals as top electrodes for perovskite solar cells. The structure used in the study was FTO/mp-TiO_2_/Perovskite/Spiro-OMetad/Metal. The metals used were Au, Ag, Pt, Ni, Cu, and Cr, with the efficiency values obtained for these metals being 16.4%, 16.25%, 14.51%, 7.30%, 8.79%, and 0.05%, respectively [35].

Samantaray et al. investigated the use of Au and Cu metals as top electrodes for perovskite solar cells and evaluated their stability after 50 days. In the study, it was reported that 5.61% efficiency was achieved when Cu was used and 5.92% efficiency was achieved when Au was used in cells with 2 cm^2^ active area. After 50 days, devices using Au had 74% of their initial efficiency, while this value dropped to 35% in devices using Cu [43].

Although polymer solar cells have been widely investigated due to their low-cost potential, the choice of electrode materials remains a critical factor affecting both device performance and long-term stability. To the best of our knowledge, the use of Cu as top contact in polymer solar cells has not been previously reported. However, Cu is also known to be more susceptible to oxidation and interfacial degradation, particularly in the presence of O_2_ and moisture, which are among the most critical factors limiting the operational stability of polymer solar cells. In this context, the present study systematically investigates the feasibility of employing Cu as a top electrode by focusing not only on device efficiency, but also on the long-term stability and degradation mechanisms. Stability tests were conducted over 12 weeks under different environmental conditions, including room conditions, desiccator, and glovebox environments, in order to elucidate the role of ambient factors on device degradation and to assess whether Cu can serve as a cost-effective and reliable alternative to conventional noble metal electrodes.

## 2. Materials and Methods

### 2.1. Materials

The chemicals used did not undergo any purification process. Indium-doped tin oxide (ITO)-coated glasses (15 Ω/square) used as a substrate in the solar cell were purchased from Kintec (Kowloon, Hong Kong), and acetone (99.5%) and isopropyl alcohol (99.5%) used for cleaning were purchased from Tekkim (Bursa, Türkiye). The Zn(CH_3_COO)_2_·2H_2_O (98–101%) and 2-Methoxyethanol (99%) used in the sol–gel method were supplied by Alfa Aesar (Karlsruhe, Germany), and ethanolamine (99.5%) was supplied by Aldrich (St. Louis, MO, USA).

Poly(3-hexylthiophene-2,5-diyl) (P3HT) (93%) and (6,6)-phenyl C61 butyric acid methyl ester (PCBM) (99.5%), which constitute the active layer of the solar cell, were obtained from Lum-Tech (New Taipei City, Taiwan). The hole transport layer of the solar cell, composed of MoO_3_ (99.8%), was procured from Sigma-Aldrich (St. Louis, MO, USA).

### 2.2. Methods

Commercially purchased ITOs were cleaned in an ultrasonic bath with distilled water, acetone, and isopropyl alcohol. Then, the ZnO layer, which is explained in detail below, was coated. Just before the ZnO coating, the ITOs were kept in the UV-O_3_ cleaner for 20 min to both clean any organic residues and make the surface more hydrophilic.

The method recommended by Liang et al. was used for ZnO synthesized by the sol–gel method [44]. In this method, first, 2-methoxy ethanol is added to Zn(CH_3_COO)_2_·2H_2_O and mixing is applied with temperature. When the temperature reaches approximately 60 °C, monoethanol amine (MEA) is added. The molar ratios of MEA and Zn(CH_3_COO)_2_·2H_2_O are kept at 1:1. After stirring for 2 h at 60 °C, the reaction is terminated and left to age for 1 day. After aging, the solution is ready for coating. The prepared ZnO solution was coated on ITO with the spin coater at 2000 rpm for 30 s. The coated films were annealed at 150 °C for 30 min. ZnO thin films were coated and annealed in air.

The active layer was prepared by mixing P3HT:PCBM (10 mg:8 mg) in 1 mL chlorobenzene at 60 °C overnight. The prepared mixture was coated on ZnO with a spin-coater at 1250 rpm for 40 s and annealed at 120 °C for 20 min in a glovebox. MoO_3_ was coated on the active layer with a thickness of 4 nm at 10^−6^ torr pressure by the physical vapor deposition (PVD) method. Finally, 80 nm Cu was coated using PVD.

### 2.3. Characterizations

**Figure 1:**
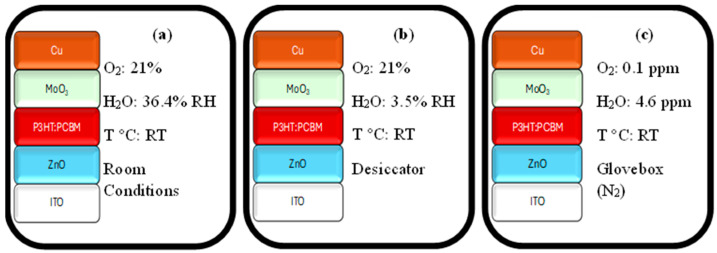
Schematic representation of specific test conditions for each environment. (**a**) Room conditions. (**b**) Desiccator. (**c**) Glovebox.

The efficiency of devices was measured with a Keithley 2400 (Keithley Instruments, Cleveland, OH, USA) source meter, OAI Trisol (class AAA, 1000 W, San Jose, CA, USA), coupled with an AM1.5 filter, which was used as the light source, and a light intensity of a 100 mW/cm^2^, used in all measurements. The electrical characterization of all devices was conducted in the glovebox. The devices were kept in different environments (room conditions, desiccator, and glovebox) for 12 weeks, and the effects of O_2_ and humidity on the stability of the devices were investigated. The specific test conditions for room conditions, desiccator, and glovebox environments are provided in Figure 1. No intervention was made regarding ambient temperatures during the 12-week period (Room Temperature (RT)). The relative humidity (RH) of the desiccator and room conditions were determined (3.5% and 36.4%) using a Testo 440 humidity meter (Titisee-Neustadt, Germany). For both the desiccator and ambient conditions, the O_2_ concentration was assumed to be equivalent to that of normal atmospheric levels (21%). The glovebox atmosphere was filled with nitrogen (N_2_), with O_2_ and moisture levels maintained at 0.1 ppm and 4.6 ppm, respectively.

The Cu-coated devices were characterized by XRD (Bruker D8 Advance, Karlsruhe, Germany) using CuKα radiation (λKα = 1.5405 Å) to determine whether CuO_x_ phases were formed. In addition, FTIR (Bruker Vertex 70, Billerica, MA, USA) analyses were performed to investigate the possible formation of O_2−_ and moisture-related species on the surface of the Cu contacts.

## 3. Results and Discussion

**Figure 2:**
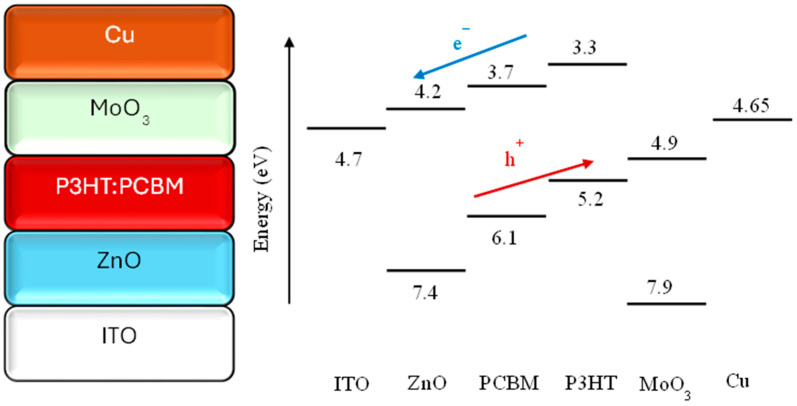
Schematic representation of devices and their energy levels [34,36,45,46].

The main motivation for this study is the low cost of Cu metal. On December 2024, the price of Cu per gram was approximately 0.01 USD at the London Metal Exchange, while the price of Ag per gram was approximately 1 USD at the London Bullion Market Association [38,39]. However, the fact that a material has a low cost alone is not enough to support its use. The compatibility of the highest-occupied molecular orbital (HOMO)–lowest-unoccupied molecular orbital (LUMO) levels and work functions of the materials used in a device’s design is very important. The schematic representation and energy levels of the produced devices are shown in Figure 2 [34,36,45,46]. According to Figure 2, it can be seen that Cu is perfectly compatible for this device’s structure. Additionally, as shown in Table 1, Cu is one of the metals with the best electrical conductivity.

In this study, firstly, it was determined whether competitive efficiency values could be achieved when Cu was used as the top contact. The highest efficiency achieved among the produced devices was 2.86%. The electrical parameters of this device were measured as 6.9 mA/cm^2^ (J_sc_), 610 mV (V_oc_), and 68% (FF). These results are acceptable for P3HT:PCBM-based polymer solar cells. In particular, the V_oc_ and FF values are adequate. In the next part of the study, stability tests on the produced devices were carried out depending on different atmospheric conditions (room conditions, desiccator, and glovebox). Thus, the effects of both moisture and O_2_ on the devices were investigated separately. Stability tests were performed for 12 weeks. Table 2 shows the time-dependent changes in the devices that provide the highest efficiency. In Table 3, the time-dependent changes in the devices with the highest efficiency are presented after normalization. Table 4 shows the average values of the produced devices. In Table 5, the averages of the time-dependent changes in the produced devices are given normalized. In addition to efficiency, the other electrical parameters used in efficiency calculations are also included in the tables.

The study started by producing nine devices for each environment. However, undesirable problems occurred in some devices over the 12-week period. Therefore, the reported results are based on the averages of five devices for both the glovebox and room conditions and three devices for the desiccator.

In addition to these results, to evaluate Cu and Ag not only in terms of cost, but also in terms of stability, the results of the 8-week stability tests previously conducted in our study are presented in Tables S1 and S2. The referenced study also employed the same device architecture (ITO/ZnO/P3HT:PCBM/MoO_3_/Metal) used in the present work [47]. The only difference is the use of Ag instead of Cu as the top contact. Table 1 shows the normalized results of the best-performing devices, while Table S2 presents the normalized average results of all fabricated devices. Table S2 was calculated using the data obtained from the Supporting Information section of the referenced article. When the normalized average performance values of the devices over the 8-week period are compared, it is observed that the efficiency values under room conditions are relatively similar. However, devices stored in the desiccator clearly show that Ag provides much more stable results. Considering that the desiccator environment is almost free of humidity and that the main degradation mechanism is likely driven by the presence of O_2_, the greater stability of Ag compared to Cu is an expected outcome.

A comparison of the T_80_ values of devices employing Ag and Cu metals is presented in Table 6. It should be noted that, since the tests conducted with Ag covered only an 8-week period, the exact T_80_ value could not be determined. Therefore, it is reported as +1344 h. Here, T_80_ denotes the time at which the device efficiency decreases to 80% of its initial value. A striking point emerges when evaluating the glovebox conditions by comparing the best-performing devices. This indicates that when the contact of the devices with O_2_ and moisture is eliminated, Cu can function effectively as an electrode.

**Figure 3:**
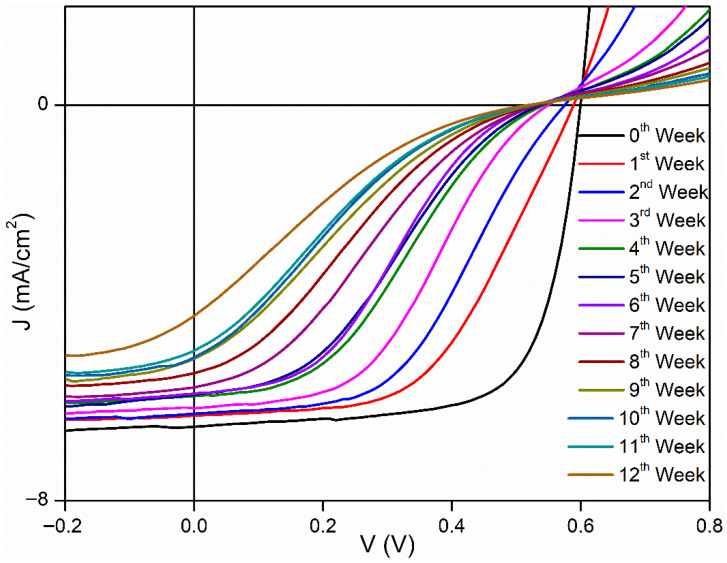
Time-dependent J–V graphs of best devices kept under room conditions.

**Figure 4:**
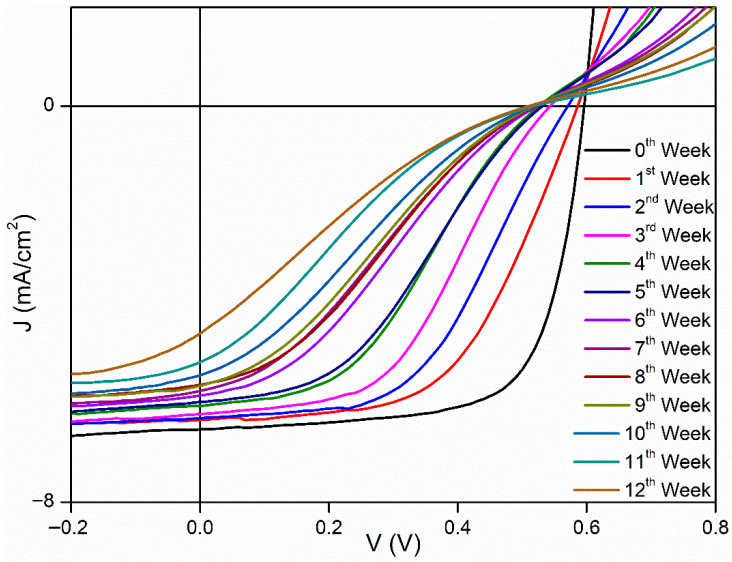
Time-dependent J–V graphs of best devices kept in desiccator.

**Figure 5:**
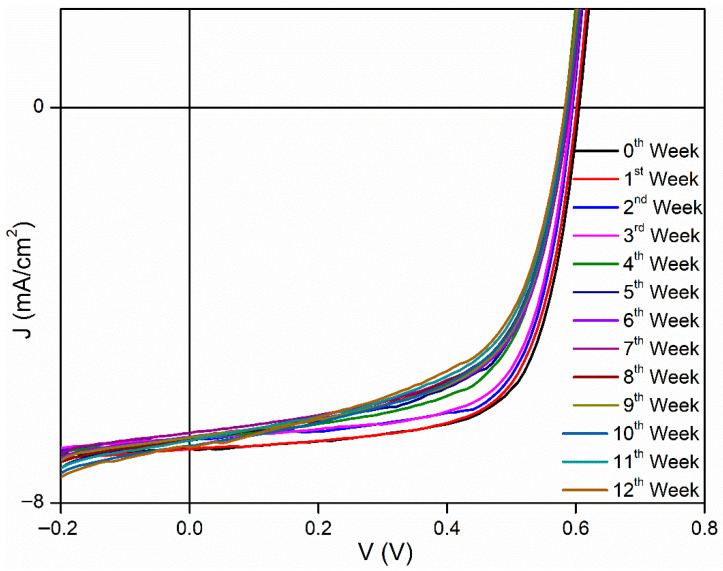
Time-dependent J–V graphs of best devices kept in glovebox.

Figure 3, Figure 4 and Figure 5 show J–V graphs of the time-dependent changes in the best devices kept in different environments. By examining Table 1, the time-dependent changes in the electrical parameters of the devices that provide the highest efficiency for each environment can be observed. For the room conditions, the initial values obtained were 6.51 mA/cm^2^ (J_sc_), 610 mV (V_oc_), 69.3% (FF), and 2.71% (η). By the end of the 12th week, these values had decreased to 4.26 mA/cm^2^ (J_sc_), 520 mV (V_oc_), 17.9% (FF), and 0.4% (η). The electrical values of the best device stored in the desiccator were initially 6.53 mA/cm^2^ (J_sc_), 600 mV (V_oc_), 68.7% (FF), and 2.69% (η). By the end of the 12th week, these values had decreased to 4.59 mA/cm^2^ (J_sc_), 510 mV (V_oc_), 20.7% (FF), and 0.49% (η). Under glovebox conditions, the values recorded at the beginning were 6.9 mA/cm^2^, 610 mV, 68%, and 2.86%. After 12 weeks, these values had dropped to 6.87 mA/cm^2^, 580 mV, 68%, and 2.86%. To facilitate a better understanding of these values, the results have been normalized and are presented in Table 3. As seen in Table 3, the time-dependent decreases in the electrical parameters of the devices stored under glovebox, desiccator, and room conditions are as follows: for J_sc_, a reduction of 1%, 30%, and 35%; for V_oc_, 5%, 15%, and 13%; for FF, 17%, 70%, and 74%; and for η, 22%, 82%, and 85%, respectively.

**Figure 6:**
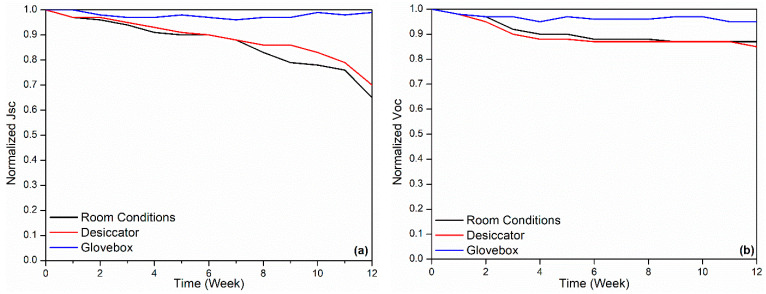

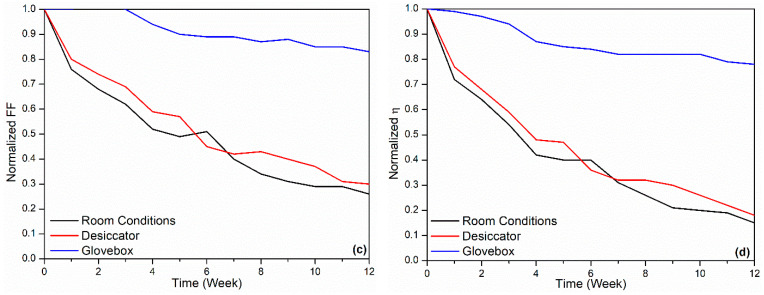
(**a**) Time-normalized J_sc_, (**b**) time-normalized V_oc_, (**c**) time-normalized FF, (**d**) time-normalized efficiency graphs of best devices.

**Figure 7:**
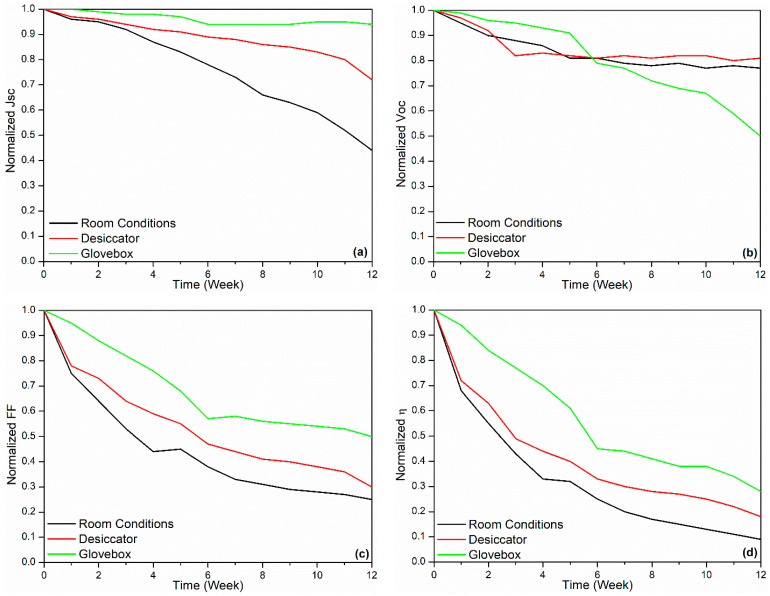
(**a**) Time-normalized J_sc_, (**b**) time-normalized V_oc_, (**c**) time-normalized FF, (**d**) time-normalized efficiency graphs of average devices.

The results of the best device kept in the glovebox are quite promising. There was only a 1% decrease in current density and only a 5% decrease in V_oc_. The decrease in efficiency is mainly due to FF. It should be noted that the values mentioned here are the time-dependent changes in the devices with the highest efficiency. The averages of all devices are given in Table 4 and Table 5. Additionally, the results presented in Table 3 and Table 5 are shown graphically in Figure 6 and Figure 7.

The V_oc_ of the devices is related to the energy levels of the materials that form the active layer. In particular, no significant change in V_oc_ was observed for the best devices. This indicates that P3HT and PCBM did not undergo noticeable degradation [48].

Upon examining Table 5, it can be observed that by the end of the 12th week, the decrease in J_sc_ was 56% under room conditions, while it was 28% in the desiccator. Considering that there is no moisture in the desiccator, only interaction with O_2_ occurs in this environment. Under room conditions, both O_2_ and moisture interact with the device. Therefore, the decrease in J_sc_ appears to be equally influenced by both O_2_ and moisture. The decrease in current density can be attributed to an increase in series resistance. Furthermore, interfacial degradation involving the buffer layers may promote additional recombination pathways at both the active layer and contact interfaces [48]. By analyzing the FF values, 75% decrease is observed under room conditions, while in the desiccator, this value is 70%. From here, it can be concluded that the main condition affecting FF is O_2_ because, while the effect of O_2_ is seen to be 70%, the effect of humidity is only 5%. The situation with V_oc_ is similar to that of FF. The decrease due to O_2_ is 19%, while the decrease caused by moisture is only 4%.

Additionally, two of the devices subjected to stability tests under glovebox conditions showed a greater than expected decline. This reduction, particularly noticeable from the 6th week onwards, primarily affected the V_oc_ values. It is believed that this occurred due to scratches on the devices during measurement, which interacted with O_2_ and moisture when the devices were taken out of the glovebox for photography. At this stage, oxidation may have occurred on the Cu surface in some devices due to scratches that allowed for contact with O_2_. Furthermore, O_2_ diffusion through these scratches may have reached the active layer, leading to possible alterations in its HOMO-LUMO energy levels. The most significant factor influencing the V_oc_ of the devices is the difference between the HOMO level of the donor and the LUMO level of the acceptor materials. If the devices had not been taken out from the glovebox for even the photo capture, it is expected that the decrease in V_oc_ would have been minimal (such as for the devices with the highest efficiency).

**Figure 8:**
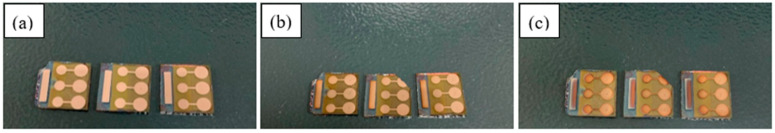
Photographs of the devices stored under room conditions at different time intervals: (**a**) 1st week, (**b**) 6th week, (**c**) 12th week.

**Figure 9:**
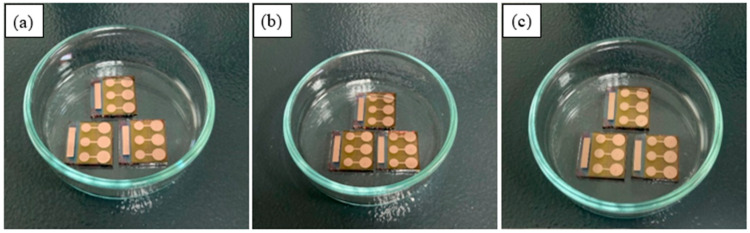
Photographs of the devices stored under desiccator at different time intervals: (**a**) 1st week (**b**) 6th week, (**c**) 12th week.

**Figure 10:**
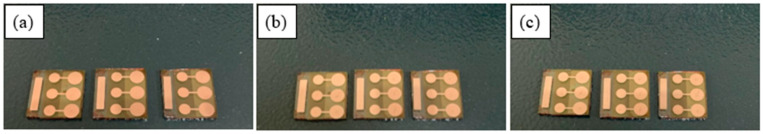
Photographs of the devices stored under glovebox at different time intervals: (**a**) 1st week (**b**) 6th week, (**c**) 12th week.

Finally, Figure 8, Figure 9 and Figure 10 show photographs of the devices stored under different conditions at various time intervals. In each figure, Figure 8a–c, Figure 9a–c and Figure 10a–c correspond to the photographs taken at the end of the 1st, 6th, and 12th weeks, respectively. In Figure 8c, by the end of the 12th week, the damage caused by O_2_ and the moisture on the devices stored under room conditions is clearly visible. It is generally accepted that O_2_ and moisture diffuse across the metal boundary and through the pinholes to the inner interface of the electrode. As a result of this diffusion, a chemical reaction takes place between the metal and O_2_ and/or the moisture. These reactions lead to mechanical breakdown, delamination, and the formation of insulating layers [49]. Although these photographs are evidence of degradation on the Cu electrode, it should not be forgotten that there are other degradation mechanisms in polymer solar cells. These mechanisms are the degradation of electrodes, electrode–active layer interfaces, and the active layer [49]. The degradation of the electrode–active layer interface can be prevented using buffer layers. However, in this case, defects may occur at the electrode–buffer layers [47].

To elucidate the degradation mechanisms of the devices stored under different environmental conditions, XRD and FTIR analyses were performed. The XRD patterns are presented in Figure S1. Based on the storage conditions, the formation of CuO or Cu_2_O phases was expected for the devices kept under ambient room conditions and in the desiccator. However, a broad diffraction peak originating from the glass substrate in the 25–35° range may have suppressed the characteristic peaks of CuO or Cu_2_O. In addition, the diffraction peaks associated with ITO may also overlap with and mask the expected oxide peaks. Specifically, the characteristic peaks of ITO appear at approximately 30.5° and 35.5°, while those of CuO are located at around 32.5° and 35.5° and those of Cu_2_O at approximately 29.5° and 36.5°.

Furthermore, it is possible that only a very small amount of CuO or Cu_2_O was formed, causing the corresponding diffraction signals to remain below the detection limit of XRD. Upon examining Figure S1, it is evident that the diffraction pattern is consistent with the face-centered cubic (FCC) crystal structure of Cu, in accordance with PDF card no. 04–0836. This observation indicates that the Cu at the surface was not fully oxidized. Notably, however, a gradual decrease in the intensity of the characteristic Cu diffraction peaks is observed when moving from glovebox conditions to ambient room conditions. This trend suggests the possible formation of new interfacial or intermediate phases during exposure to O_2_ and moisture.

**Figure 11:**
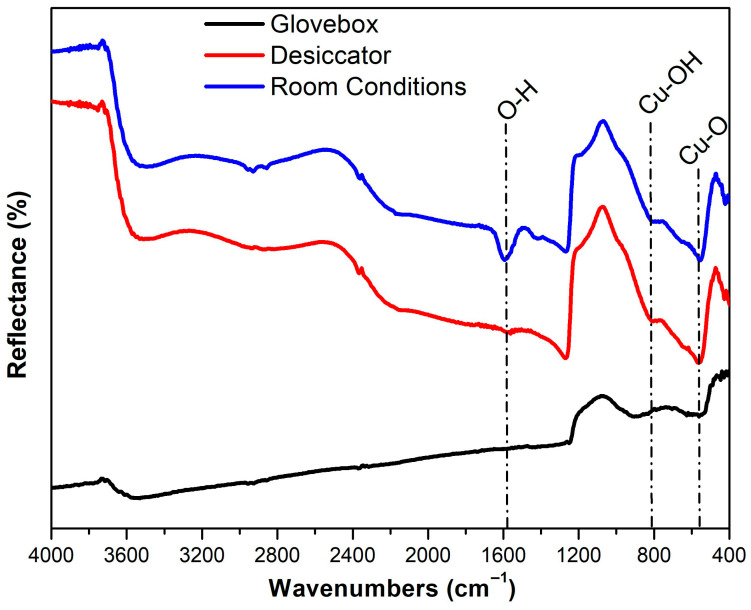
FTIR spectrum of devices with Cu electrodes stored in different environments.

FTIR analyses were performed to examine the possible formation of O_2_- and moisture-related chemical species on the Cu electrode’s surface, and the results are presented in Figure 11.

Upon examination of Figure 11, the peak observed at approximately 1590 cm^−1^ is assigned to O–H bending vibrations [50,51], the peak around 820 cm^−1^ is associated with Cu-OH bonding [52], and the peak near 550 cm^−1^ corresponds to Cu-O vibrational modes [53,54]. The band at ~1590 cm^−1^ originates from moisture-related O-H vibrations. Its absence in devices stored under glovebox conditions, very weak presence in desiccator-stored devices, and pronounced appearance in devices kept under ambient room conditions clearly indicate its correlation with moisture exposure.

The peak observed around 820 cm^−1^ is related to -OH groups that are bonded to the Cu surface, and its absence under glovebox conditions further supports this interpretation. Similarly, the Cu-O vibrational band near 550 cm^−1^ is not observed in glovebox-stored devices, but appears in those aged under desiccator and ambient room conditions, which is indicative of oxidation processes occurring at the Cu surface.

Although O_2_ and humidity are known to be major external factors affecting the stability of polymer solar cells, the chemical characteristics of the top electrode material also play a critical role in device degradation. Unlike Ag, Cu is more prone to oxidation, leading to the formation of CuO/Cu_2_O under ambient conditions. The formation of these oxide species at the MoO_3_/Cu interface can increase the contact resistance and hinder efficient hole extraction, resulting in a pronounced decrease in J_sc_ and FF. Therefore, the observed performance degradation in Cu-based devices is not solely governed by O_2_ and moisture exposure, but is also strongly influenced by the intrinsic chemical reactivity of the Cu electrode and its interfacial interactions.

Table 7 summarizes representative studies reporting the use of Cu and other metals as electrodes in different types of solar cells, highlighting the electrode’s position in comparison with the present work.

Based on our calculations under the conditions used in this study, the use of a Cu electrode provides an approximately 10% cost advantage per cell compared to a Ag electrode. Moreover, when transitioning to industrial-scale production, replacing vacuum-based processes with solution processing is expected to further enhance this cost advantage. Beyond cost considerations, another important factor is material availability. Cu is approximately 1000 times more abundant in the Earth’s crust than Ag [55]. Therefore, from a commercialization perspective, Cu emerges as a highly promising electrode candidate due to its low cost, abundance, and high electrical conductivity.

## 4. Conclusions

In this work, inverted polymer solar cells employing Cu as the top electrode were fabricated and systematically evaluated to assess their performance, stability, and cost-effectiveness. The best-performing device achieved an efficiency (η) of 2.86%, with favorable V_oc_ and FF values, indicating that Cu is a suitable electrode material for polymer solar cells.

In addition to device performance, the operational stability of the Cu-based devices was investigated under glovebox, desiccator, and ambient room conditions. The stability tests revealed that device degradation is strongly influenced by exposure to O_2_ and moisture. While devices stored under ambient and desiccator conditions exhibited pronounced efficiency losses, those maintained under glovebox conditions retained approximately 78% of their initial efficiency after 12 weeks.

From a commercialization perspective, the substantially lower cost, high electrical conductivity, and greater elemental abundance of Cu make it a promising electrode material for polymer solar cells, provided that effective encapsulation strategies are employed. Future studies should focus on evaluating Cu electrodes in high-efficiency non-fullerene systems and under combined thermal and light-induced stress conditions to further assess their long-term operational stability.

## Figures and Tables

**Table 1:** Electrical resistivity and conductivity of commonly used metal electrodes at room temperature [40].

Metal	Resistivity, *ρ* (µΩ·cm)	Conductivity, σ (S·cm^−1^)
Al	2.709	3.69 × 10^5^
Au	2.255	4.43 × 10^5^
Ag	1.617	6.18 × 10^5^
Cu	1.712	5.84 × 10^5^

**Table 2:** Time-dependent electrical parameters of best solar cells kept under different conditions.

Time (Week)	Room Conditions				Desiccator				Glovebox			
	J_sc_ (mA/cm^2^)	V_oc_ (mV)	FF (%)	η (%)	J_sc_ (mA/cm^2^)	V_oc_ (mV)	FF (%)	η (%)	J_sc_ (mA/cm^2^)	V_oc_ (mV)	FF (%)	η (%)
0	6.51	600	69.3	2.71	6.53	600	68.7	2.69	6.90	610	68.0	2.86
1	6.28	590	52.7	1.95	6.34	590	55.1	2.06	6.91	600	67.9	2.82
2	6.24	580	47.4	1.72	6.31	570	50.6	1.82	6.73	590	69.5	2.76
3	6.13	550	43.0	1.45	6.22	540	47.3	1.59	6.70	590	68.0	2.69
4	5.89	540	36.2	1.15	6.06	530	40.2	1.29	6.71	580	63.9	2.49
5	5.85	540	33.7	1.07	5.97	530	39.3	1.25	6.74	590	61.2	2.43
6	5.84	530	35.1	1.09	5.85	520	31.2	0.95	6.66	590	60.7	2.39
7	5.71	530	27.6	0.84	5.75	520	28.6	0.86	6.59	590	60.2	2.34
8	5.42	530	23.8	0.69	5.63	520	29.8	0.87	6.72	590	59.0	2.34
9	5.13	520	21.5	0.57	5.65	520	27.6	0.81	6.67	590	60.1	2.37
10	5.10	520	20.2	0.54	5.44	520	25.2	0.71	6.84	590	57.1	2.34
11	4.97	520	19.9	0.51	5.17	520	21.4	0.58	6.74	580	58.2	2.27
12	4.26	520	17.9	0.40	4.59	510	20.7	0.49	6.87	580	56.2	2.24

**Table 3:** Time-dependent electrical parameters of normalized best solar cells kept under different conditions.

Time (Week)	Room Conditions				Desiccator				Glovebox			
	J_sc_ (mA/cm^2^)	V_oc_ (mV)	FF (%)	η (%)	J_sc_ (mA/cm^2^)	V_oc_ (mV)	FF (%)	η (%)	J_sc_ (mA/cm^2^)	V_oc_ (mV)	FF (%)	η (%)
0	1	1	1	1	1	1	1	1	1	1	1	1
1	0.97	0.98	0.76	0.72	0.97	0.98	0.8	0.77	1	0.98	1	0.99
2	0.96	0.97	0.68	0.64	0.97	0.95	0.74	0.68	0.98	0.97	1.02	0.97
3	0.94	0.92	0.62	0.54	0.95	0.90	0.69	0.59	0.97	0.97	1	0.94
4	0.91	0.90	0.52	0.42	0.93	0.88	0.59	0.48	0.97	0.95	0.94	0.87
5	0.90	0.90	0.49	0.40	0.91	0.88	0.57	0.47	0.98	0.97	0.90	0.85
6	0.90	0.88	0.51	0.40	0.90	0.87	0.45	0.36	0.97	0.96	0.89	0.84
7	0.88	0.88	0.40	0.31	0.88	0.87	0.42	0.32	0.96	0.96	0.89	0.82
8	0.83	0.88	0.34	0.26	0.86	0.87	0.43	0.32	0.97	0.96	0.87	0.82
9	0.79	0.87	0.31	0.21	0.86	0.87	0.40	0.30	0.97	0.97	0.88	0.82
10	0.78	0.87	0.29	0.20	0.83	0.87	0.37	0.26	0.99	0.97	0.85	0.82
11	0.76	0.87	0.29	0.19	0.79	0.87	0.31	0.22	0.98	0.95	0.85	0.79
12	0.65	0.87	0.26	0.15	0.70	0.85	0.30	0.18	0.99	0.95	0.83	0.78

**Table 4:** Time-dependent averaged electrical parameters of solar cells kept under different conditions.

Time (Week)	Room Conditions				Desiccator				Glovebox			
	J_sc_ (mA/cm^2^)	V_oc_ (mV)	FF (%)	η (%)	J_sc_ (mA/cm^2^)	V_oc_ (mV)	FF (%)	η (%)	J_sc_ (mA/cm^2^)	V_oc_ (mV)	FF (%)	η (%)
0	6.47 ± 0.04	594 ± 5.48	64.8 ± 3.65	2.50 ± 0.18	6.5 ± 0.33	600 ± 0	67.2 ± 3.22	2.66 ± 0.05	6.85 ± 0.07	602 ± 4.48	67.1 ± 2.43	2.76 ± 0.13
1	6.20 ± 0.08	562 ± 19.2	48.8 ± 3.64	1.70 ± 0.18	6.33 ± 0.14	580 ± 10.0	52.4 ± 2.44	1.92 ± 0.13	6.85 ± 0.12	594 ± 8.94	64.0 ± 4.12	2.60 ± 0.23
2	6.14 ± 0.09	532 ± 40.9	41.2 ± 6.96	1.36 ± 0.33	6.23 ± 0.09	550 ± 26.5	48.9 ± 3.79	1.67 ± 0.15	6.76 ± 0.06	580 ± 10	58.9 ± 7.50	2.32 ± 0.32
3	5.91 ± 0.21	520 ± 30.0	34.6 ± 8.72	1.08 ± 0.34	6.10 ± 0.11	493 ± 80.8	43.0 ± 3.93	1.31 ± 0.33	6.73 ± 0.10	570 ± 23.5	55.1 ± 9.83	2.13 ± 0.46
4	5.63 ± 0.28	510 ± 26.5	28.5 ± 7.86	0.83 ± 0.30	5.99 ± 3.14	497 ± 66.6	39.6 ± 3.14	1.17 ± 0.11	6.73 ± 0.12	562 ± 14.8	50.9 ± 7.48	1.93 ± 0.33
5	5.39 ± 0.76	480 ± 61.2	29.2 ± 6.83	0.79 ± 0.31	5.89 ± 0.07	493 ± 63.5	37.0 ± 2.72	1.08 ± 0.17	6.62 ± 0.20	550 ± 29.2	46.0 ± 9.90	1.70 ± 0.49
6	5.08 ± 0.86	480 ± 52.0	24.9 ± 6.74	0.63 ± 0.30	5.77 ± 0.12	487 ± 66.6	31.5 ± 5.56	0.87 ± 0.09	6.45 ± 0.12	474 ± 94.5	38.6 ± 12.6	1.24 ± 0.67
7	4.74 ± 0.96	468 ± 64.6	21.7 ± 4.98	0.50 ± 0.23	5.72 ± 0.03	497 ± 60.8	29.1 ± 2.94	0.79 ± 0.04	6.43 ± 0.17	462 ± 104	38.7 ± 12.3	1.21 ± 0.67
8	4.31 ± 1.16	462 ± 70.7	20.1 ± 3.81	0.42 ± 0.20	5.55 ± 0.07	483 ± 63.5	27.8 ± 3.04	0.74 ± 0.11	6.50 ± 0.16	434 ± 131	37.3 ± 12.4	1.13 ± 0.72
9	4.01 ± 1.17	468 ± 63.8	19.0 ± 3.13	0.37 ± 0.17	5.50 ± 0.13	493 ± 46.2	26.6 ± 2.36	0.72 ± 0.08	6.43 ± 0.15	416 ± 129	36.9 ± 13.1	1.07 ± 0.76
10	3.79 ± 1.15	460 ± 67.6	18.0 ± 2.80	0.33 ± 0.16	5.40 ± 0.06	490 ± 52.0	25.1 ± 2.50	0.66 ± 0.04	6.52 ± 0.21	402 ± 135	36.2 ± 12.3	1.04 ± 0.76
11	3.38 ± 1.32	462 ± 69.8	17.6 ± 2.64	0.29 ± 0.16	5.20 ± 0.16	480 ± 60.8	23.7 ± 3.54	0.58 ± 0.02	6.52 ± 0.15	358 ± 159	35.2 ± 13.0	0.93 ± 0.79
12	2.85 ± 1.23	466 ± 56.3	15.8 ± 1.82	0.22 ± 0.15	4.69 ± 0.11	490 ± 34.6	20.3 ± 0.56	0.47 ± 0.03	6.50 ± 0.24	300 ± 171	33.2 ± 13.0	0.77 ± 0.84

**Table 5:** Time-dependent averaged electrical parameters of normalized solar cells kept under different conditions.

Time (Week)	Room Conditions				Desiccator				Glovebox			
	J_sc_ (mA/cm^2^)	V_oc_ (mV)	FF (%)	η (%)	J_sc_ (mA/cm^2^)	V_oc_ (mV)	FF (%)	η (%)	J_sc_ (mA/cm^2^)	V_oc_ (mV)	FF (%)	η (%)
0	1	1	1	1	1	1	1	1	1	1	1	1
1	0.96 ± 0.01	0.95 ± 0.03	0.75 ± 0.06	0.68 ± 0.07	0.97 ± 0.03	0.97 ± 0.02	0.78 ± 0.04	0.72 ± 0.05	1 ± 0.01	0.99 ± 0.01	0.95 ± 0.06	0.94 ± 0.06
2	0.95 ± 0.01	0.90 ± 0.06	0.64 ± 0.11	0.55 ± 0.13	0.96 ± 0.04	0.92 ± 0.04	0.73 ± 0.09	0.63 ± 0.06	0.99 ± 0.01	0.96 ± 0.01	0.88 ± 0.10	0.84 ± 0.10
3	0.92 ± 0.03	0.88 ± 0.05	0.53 ± 0.18	0.43 ± 0.14	0.94 ± 0.05	0.82 ± 0.13	0.64 ± 0.04	0.49 ± 0.11	0.98 ± 0.01	0.95 ± 0.04	0.82 ± 0.14	0.77 ± 0.15
4	0.87 ± 0.04	0.86 ± 0.04	0.44 ± 0.13	0.33 ± 0.11	0.92 ± 0.04	0.83 ± 0.11	0.59 ± 0.08	0.44 ± 0.04	0.98 ± 0.01	0.93 ± 0.02	0.76 ± 0.11	0.70 ± 0.10
5	0.83 ± 0.12	0.81 ± 0.10	0.45 ± 0.11	0.32 ± 0.12	0.91 ± 0.05	0.82 ± 0.10	0.55 ± 0.05	0.4 ± 0.06	0.97 ± 0.02	0.91 ± 0.04	0.68 ± 0.14	0.61 ± 0.15
6	0.78 ± 0.13	0.81 ± 0.08	0.38 ± 0.09	0.25 ± 0.10	0.89 ± 0.03	0.81 ± 0.11	0.47 ± 0.11	0.33 ± 0.04	0.94 ± 0.02	0.79 ± 0.15	0.57 ± 0.18	0.45 ± 0.23
7	0.73 ± 0.15	0.79 ± 0.11	0.33 ± 0.07	0.20 ± 0.08	0.88 ± 0.04	0.82 ± 0.10	0.44 ± 0.07	0.3 ± 0.02	0.94 ± 0.02	0.77 ± 0.17	0.58 ± 0.18	0.44 ± 0.23
8	0.66 ± 0.18	0.78 ± 0.11	0.31 ± 0.05	0.17 ± 0.08	0.86 ± 0.05	0.81 ± 0.11	0.41 ± 0.06	0.28 ± 0.04	0.94 ± 0.02	0.72 ± 0.21	0.56 ± 0.18	0.41 ± 0.25
9	0.63 ± 0.19	0.79 ± 0.10	0.29 ± 0.05	0.15 ± 0.06	0.85 ± 0.04	0.82 ± 0.08	0.40 ± 0.06	0.27 ± 0.03	0.94 ± 0.02	0.69 ± 0.21	0.55 ± 0.19	0.38 ± 0.26
10	0.59 ± 0.17	0.77 ± 0.11	0.28 ± 0.04	0.13 ± 0.06	0.83 ± 0.04	0.82 ± 0.09	0.38 ± 0.06	0.25 ± 0.02	0.95 ± 0.03	0.67 ± 0.22	0.54 ± 0.18	0.38 ± 0.26
11	0.52 ± 0.20	0.78 ± 0.12	0.27 ± 0.04	0.11 ± 0.06	0.80 ± 0.02	0.80 ± 0.10	0.36 ± 0.07	0.22 ± 0.02	0.95 ± 0.03	0.59 ± 0.26	0.53 ± 0.18	0.34 ± 0.27
12	0.44 ± 0.18	0.79 ± 0.09	0.25 ± 0.01	0.09 ± 0.05	0.72 ± 0.05	0.81 ± 0.06	0.30 ± 0.02	0.18 ± 0.01	0.94 ± 0.03	0.50 ± 0.28	0.50 ± 0.19	0.28 ± 0.29

**Table 6:** Comparison of T_80_ values of best devices using Ag and Cu.

	Room Conditions	Desiccator	Glovebox	Reference
Ag	~408 h	~270 h	+1344 h	[47]
Cu	~120 h	~146 h	~1792 h	This work

**Table 7:** Summary of representative studies on the use of Cu and other metals as electrodes in solar cells.

Solar Cell Type	Investigated Metal	Electrode Position	Reference
Perovskite Solar Cell	Au, Ag, Pt, Ni, Cu, Cr	Top	[35]
Polymer Solar Cell	Al, Ag, Au	Top	[41]
Polymer Solar Cell	Au, Ag, Cu	Bottom	[42]
Perovskite Solar Cell	Au, Cu	Top	[43]
Polymer Solar Cell	Cu	Top	This work

## Data Availability

The raw data supporting the conclusions of this article will be made available by the author on request.

## Supplementary Materials

The following supporting information can be downloaded at https://www.mdpi.com/article/10.3390/polym18020232/s1. Figure S1: XRD patterns of devices with Cu electrodes stored in different environments; Table S1: Stability test results of the best devices using Ag as the top contact [47], Table S2: Stability test results of the averaged devices using Ag as the top contact [47].
