# Supplementary material for: Investigation of the Use of Cu as Top Electrode in Polymer Solar Cells
Source: Polymers (Basel). 2026 Jan 16;18(2):232. doi: 10.3390/polym18020232 (PMC12845595; doi:10.3390/polym18020232)
Supplement: Supplementary file 1 [file polymers-18-00232-s001.zip › polymers-4067325-supplementary.pdf]

Investigation of the Use of Cu as Top Electrode in Polymer Solar Cells

Semih Yurtdaş

Department of Energy Systems Engineering, Faculty of Engineering, Karamanoğlu Mehmetbey University, 70100 Karaman, Türkiye
syurtdas@kmu.edu.tr

Table S1. Stability test results of the best devices using Ag as the top contact [47]

Time (Week)	Room Conditions				Desiccator				Glovebox			
	J_{sc}	V_{oc}	FF	η	J_{sc}	V_{oc}	FF	η	J_{sc}	V_{oc}	FF	η
0	1.00	1.00	1.00	1.00	1.00	1.00	1.00	1.00	1.00	1.00	1.00	1.00
1	0.94	1.00	0.94	0.89	0.93	1.00	0.90	0.83	0.97	0.98	1.00	0.96
2	0.90	1.00	0.91	0.82	0.90	1.00	0.87	0.78	0.98	1.00	0.97	0.95
3	0.89	1.00	0.86	0.77	0.88	1.00	0.84	0.75	0.99	0.98	0.98	0.95
4	0.81	0.95	0.64	0.49	0.78	0.98	0.78	0.60	0.98	0.98	0.97	0.93
6	0.66	0.72	0.47	0.22	0.71	0.98	0.75	0.52	0.95	0.98	0.95	0.89
8	0.57	0.52	0.49	0.15	0.60	0.95	0.72	0.41	0.91	0.98	0.91	0.82

Table S2. Stability test results of the averaged devices using Ag as the top contact [47]

Time (Week)	Room Conditions				Desiccator				Glovebox			
	J_{sc}	V_{oc}	FF	η	J_{sc}	V_{oc}	FF	η	J_{sc}	V_{oc}	FF	η
0	1.00	1.00	1.00	1.00	1.00	1.00	1.00	1.00	1.00	1.00	1.00	1.00
1	0.89	0.91	0.71	0.60	0.94	1.00	0.96	0.90	0.97	0.99	0.99	0.95
2	0.86	0.91	0.69	0.55	0.91	1.00	0.91	0.83	0.98	1.00	0.96	0.93
3	0.83	0.89	0.66	0.51	0.88	0.99	0.89	0.78	0.98	0.98	0.97	0.93
4	0.72	0.77	0.56	0.33	0.78	0.98	0.81	0.62	0.97	0.98	0.95	0.91
6	0.65	0.68	0.51	0.23	0.70	0.97	0.79	0.54	0.95	0.98	0.94	0.88
8	0.56	0.48	0.48	0.13	0.59	0.96	0.76	0.43	0.92	0.98	0.90	0.81

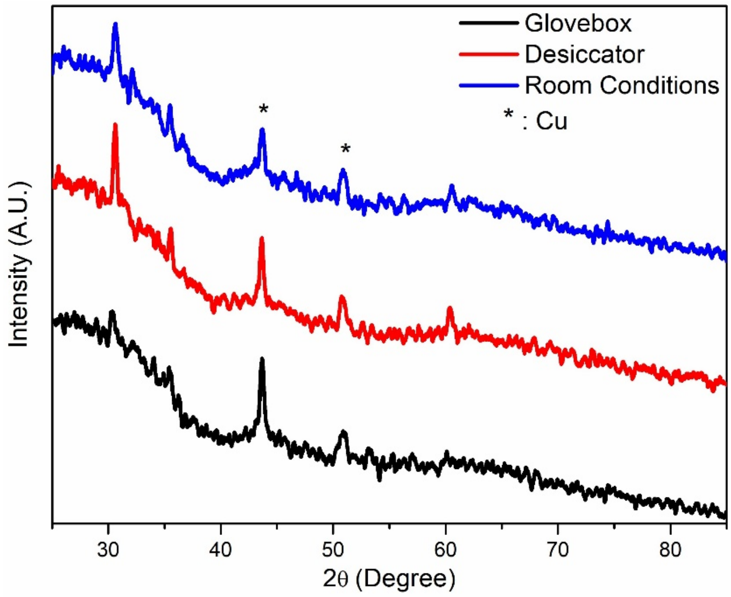

Figure S1. XRD patterns of devices with Cu electrodes stored in different environments
